# Lessons learned from the COVID pandemic and its impact on bioanalysis and drug development

**DOI:** 10.4155/bio-2021-0120

**Published:** 2021-07-19

**Authors:** Enaksha R Wickremsinhe, Catherine L Brockus, Anthony T Murphy

**Affiliations:** ^1^Eli Lilly & Company, Indianapolis, IN 46285, USA

**Keywords:** bioanalysis, clinical trials, COVID-19, COVID therapeutics, drug development, patient centric sampling, SARS-CoV-2 coronavirus

## Abstract

The COVID-19 pandemic challenged pharmaceutical and bioanalytical communities at large, in the development of vaccines and therapeutics as well as supporting ongoing drug development efforts. Existing processes were challenged to manage loss of staffing at facilities along with added workloads for COVID related study support including conducting preclinical testing, initiating clinical trials, conducting bioanalysis and interactions with regulatory agencies, all in an ultra-rapid timeframes. A key factor of success was creative rethinking of processes and removing barriers – some of which hitherto had been considered immovable. This article describes how bioanalysis was crippled at the onset of the pandemic but how innovative and highly collaborative efforts across teams within and outside of both pharma, bioanalytical labs and regulatory agencies worked together remarkably well.

The global COVID-19 pandemic has changed our lives forever. One could list some of these changes, ranging from new vocabulary (social distancing, flatten the curve), wardrobe additions (face mask), anxiety, depression and staying positive, pandemic shopping/buying (toilet paper, hand sanitizer), rampant misinformation and rumors, school closures, to the ‘new normal’ and finally, vaccines and vaccinations.

This period has challenged the entire world, without exception and has resulted in implementing safe and creative workarounds. To quote Canadian writer Robin S Sharma, *“Difficult times disrupt your conventional ways of thinking and push you to forge better habits of thought, performance and being.”* The world united to address the healthcare needs from testing to treatment to beating the virus. The scientific community embarked on a search for therapeutic and preventative solutions. Initial efforts focused on repurposing already-approved pharmacologic agents along with the development of novel therapeutics to reduce the morbidity and mortality associated with the rapidly-spreading virus. Additionally, a long-term solution was needed to prevent further spread and recurrence globally.

Immediately (almost in parallel with the first indications of its global spread), the scientific and pharmaceutical community embarked on developing, testing and producing effective therapeutics and vaccines [1,2]. To date, several vaccines have been developed, granted ‘emergency use authorization’ by global regulatory agencies and are being made available worldwide [3,4]. The successful development of these vaccines, as well as novel therapeutics targeted for the treatment of COVID-19 have been a result of an unparalleled collaboration between scientists (pharmaceutical companies), the healthcare community and the regulatory agencies – working toward a single goal of developing effective vaccines and therapies as quickly as possible to beat the SARS-CoV2 virus.

Pharmaceutical companies involved in developing either therapeutics or vaccines against the virus, had to face pandemic-related challenges and adjust accordingly to continue supporting patient enrollment in an ongoing clinical trials and the continued development of novel drugs and therapies. The remainder of this article captures the challenges and learnings related to bioanalysis and drug development.

## Priority: bioanalysis supporting COVID-19-related therapies

The global response – from developing rapid testing methods, to adoption of both current and newly developed therapeutics, to developing, testing and manufacturing vaccines faster than ever before – has been unprecedented, as captured in a recent article titled ‘How science beat the virus’ [5]. The magnitude of resources, effort and urgency to bring the pandemic under control is reflected by the number of clinical trials being conducted. A search in ClinicalTrials.gov using ‘COVID-19’ as the condition/disease resulted in 5273 studies (as of 06APR2021).

All aspects of support for these studies were happening around-the-clock, including bioanalysis and as a result, laboratories in the USA performing pharmaceutical analysis were classified as essential businesses (with essential workers). The outcomes of these efforts are evidenced by the speed at which treatments and vaccines have become available (and continue to). From a COVID-19 diagnostic testing perspective, access to testing and turnaround of test results was fraught with logistical and scientific challenges which improved over time, resulting in 24 h turnaround for several testing options (molecular/PCR-based, antigen-based and ELISA-based serology testing) [6]. The overwhelming challenge in all situations was to handle number of samples that were 1000-fold higher than what laboratories would have been accustomed to in ‘normal’ times.

## Impact on bioanalysis supporting drug development

The onslaught of studies focused on therapeutics to treat COVID-19 translated to a significant need and demand for bioanalysis to understand drug exposure and pharmacokinetics. Since bioanalytical laboratories were classified as essential businesses, they had to immediately adopt procedures to reduce risk of workers’ exposure to COVID-19, including the implementation of engineering controls, administrative controls, establishing safe work practices and providing personal protective equipment (PPE) as outlined by the Occupational Safety and Health Administration (OSHA) [7] to continue operations.

Lockdowns and implementing safe work environments created a ripple effect and caused a significant increase and urgency in the need/demand for bioanalytical support. Pharmaceutical companies laboratory capacities were significantly impacted, having to substantially reduce in-house bioanalytical support, while ongoing preclinical and clinical studies continued to generate bioanalytical samples. Resources at bioanalytical CROs became a prime commodity as many pharmaceutical companies resolved to outsource, both COVID and non-COVID related assays, to enable continued bioanalyses. Since capacity at bioanalytical CROs was also limited and further restricted due to the safety requirements necessitated due to COVID (i.e., limiting occupancy), this resulted in long wait periods (as much as 4–6 months) for placement of new non-COVID projects, which were necessarily deprioritized to make room for the influx of COVID-related work. This deprioritization effectively halted newer programs from moving into the regulated nonclinical or clinical space – both by the sponsors as well as the CROs.

## Interactions with regulatory authorities

Interactions with regulatory authorities changed dramatically as the pandemic began, and as pharma worked toward both vaccine and therapeutic treatment options. Previously unheard of timelines were requested and required in order to move potential therapies forward as rapidly as possible. Within the pharma industry, drug development timelines which might have been perceived in months to years, prepandemic, were suddenly shortened to just a few weeks or months between initiation of nonclinical studies to first human dosing. As a result, discussions with regulatory agencies took place on an almost daily basis, with decision making and collaboration based on the scientific merit of data as it arrived nearly in real time.

This collaborative effort required flexibility on the part of both pharma and the US FDA in particular, prioritization around the most critical and informative data and the ability to adapt study plans around the most relevant end points or markers without delay. In all sectors of pharma, this resulted in the most rapid response to a human disease threat ever mounted in history. Even as vaccines and therapeutics have become widely available through ‘emergency use authorizations’, additional data from clinical trials continue to be accumulated and the collaboration between pharma and the regulatory agencies remains faster paced than that experienced prepandemic.

## Impact on clinical trials & patients

Ensuring the safety of patients enrolled in clinical trials to avoid exposure to potential COVID infection was critical. Patients were typically required to return to clinical sites/physician’s offices, for multiple protocol-specified visits, including the collection of blood samples for testing (ranging from hematology and chemistry panels to biomarkers to drug concentrations for pharmacokinetic analyses). Almost immediately, access to the clinical site(s) were restricted for various reasons: the site being shut down due to COVID driven mandates by local and state governments, the shortage of PPE, concerns of safety using public transportation and patients with active COVID-19 disease. Enrollment in numerous clinical trials was put on hold worldwide as pharmaceutical companies worked to determine the best next steps in providing a means for important therapeutics to reach patients.

The FDA published a new guidance on conducting clinical trials during the COVID-19 pandemic, highlighting the need to ensure the safety of all trial participants [8]. This guidance discussed all aspects of how this pandemic can/has impacted clinical trials, ranging from quarantines and site closures, travel limitations, interruptions to the supply chain for the investigational product and other considerations if site personnel or trial participants become infected. It also addressed the handling of situations where it would not be possible to perform protocol-specified tests (i.e., collect blood samples) as well as accessing alternative sites to perform the protocol-specified tests.

Besides ensuring the safety of the patients as well as the personnel, initiation and continuation of sites were each impacted by delays related to unavailability of kits or their components and investigation product(s) due to both supply chain and shipping delays. Even when the sites were open, patients were still impacted due to COVID travel restrictions. This has prompted many trials to transform from the traditional ‘clinic-centric’ practices to new paradigms, adopting ‘patient-centric’ approaches, including the use of mobile nursing services [9]. Reworking of the logistics around patient centric sampling necessitated rethinking of sample storage and temperature control, handling, processing and delivery timelines, patient compliance in cases of self-sampling and overall sample management processes and record-keeping.

## Impact on bioanalytical operational challenges

Bioanalysis ‘begins’ upon blood sample collection from a subject/patient and continues until it progresses to the analytical detection instrument which generates a value or result. Blood sample handling may include processing to serum or plasma, separation of aliquots for specific analyses, shipment by commercial couriers to bioanalytical labs, etc., all of which requires defined steps and timing. All stages of this process can be and have been impacted by the pandemic, either due to being ‘short-handed’ in the number of employees or with issues related to supplies being delayed or limited in availability. Situations such as long delays between the drawing of blood and processing to plasma/serum, samples not being frozen or samples being shipped with insufficient dry ice can affect the integrity of the sample. In many such situations, the affected sample is not usable, which can ultimately affect the interpretation of clinical study data.

Shortages in bioanalytical lab essentials, for example, gloves, plastic tips for pipettes, polypropylene tubes and other laboratory basics, have impacted ongoing projects as well as COVID related priority projects. Globally, border closures and decline in shipping (air and sea) slowed down the supply of reagents, materials and equipment. Pipette tip manufacturers informed customers that they would only honor orders not exceeding the previous year. Additionally, manufacturing facilities were impacted, which further delayed production and delivery, as was reported with the Malaysian company Top Glove, which is one of the largest producers of gloves in the world [10]. Delays in the supply of blood sampling kits (due to limited availability of collection tubes), which are typically prepared and managed by central labs, have delayed the initiation of new trials as well as continued resupply to ongoing trials; thus, impacting committed timelines for bioanalysis and bioanalytical resources.

## Adoption of patient centric sampling

The shutdown in response to the pandemic resulted in the halting and/or delay of many ongoing clinical trials [11] and necessitated alternative approaches – transforming from being clinic centric to being patient centric. This has initiated new efforts across the pharmaceutical industry which are broadly discussed as ‘decentralized clinical trials’. Most of these efforts are gaining momentum from the alternative workflows that have resulted as a response to the pandemic. One aspect of decentralized clinical trials is the ability to collect blood samples (typically performed at a clinic by a phlebotomist) in a more decentralized location – in other words, a local laboratory or within the privacy of a patient’s home.

The pandemic necessitated access to testing that can be conducted safely, in other words, avoiding the need for travel to a hospital or clinic, resulting in drive through and at home testing options. Although most at home testing has been noninvasive (self-sampling using a nasal swab), several COVID-19 clinical trials have included in-home collection of blood samples for serology testing, using volumetric absorptive microsampling (VAMs) and Tasso blood sampling devices [12–15]. This demonstrates the utility of in-home or patient centric blood sampling devices. Although these technologies and devices have been available for several years, the pandemic has highlighted the broader applicability and need for such technologies. Laboratories are now also offering drive up phlebotomy – drive up, park, and the phlebotomist comes to you, saving the need for going in to the hospital, registering and then waiting to get blood drawn [16].

From a drug development perspective, there was an urgent and imminent need to continue ongoing trials as well as the ability to initiate new trials, safely. For most trials, especially the early phase clinical trials, the collection of blood samples to understand the drugs pharmacokinetic properties or to monitor a key biomarker corresponding to safety and/or efficacy was critical and ‘suddenly’ microsampling and patient centric sampling became a hot-topic of discussion. The need for an alternative for in-clinic phlebotomy was critical, potentially being replaced by mobile nursing for at-home blood sampling or microsampling. One could say that microsampling, despite slow uptake within the pharmaceutical industry, came out as a winner postpandemic, gaining broader adoption and popularity. Nonetheless, patient centric and microsampling techniques to collect blood samples may require additional bioanalytical resources, in other words, validation of a new assay/method and/or conducting concordance analyses between the traditional intravenous (iv.) drawn blood/plasma/serum versus the matrix collected via the patient centric or microsampling blood collection [17,18].

In the USA, significant adaptations of commercial recreation vehicles and even box trucks permitted ‘rolling laboratories’ to be constructed for some of the hardest hit populations in nursing homes. These self-contained units and support vehicles were capable of addressing not only the logistics of sample management at the patient site, with onboard centrifuges, biosafety cabinets, refrigerators and ultra-low freezers, but also PPE supplies, blood collection kits, therapeutic drug supplies and staffing. These ‘rolling laboratories’ proved to be a vital resource as not only a support to the nursing home patients and staff, but also allowed critical COVID therapeutic-related clinical trials to continue [19].

## Impact on bioanalysis

Bioanalysis, supporting all phases of drug development, are conducted under the guidelines provided by regulatory agencies and incorporates accountability from the time the sample is collected (drawing of blood) to reporting of the finalized concentration data and delivery of final reports [20,21]. Although most bioanalytical laboratories resumed a ‘new normal’ functionality, they were impacted by a range of issues: availability of key reagents, pipettes, 96-well plates (all plastic ware), etc.; the need to get things done even faster than in pre-COVID times, including rapid analysis, quality control (QC) and quality assurance (QA) reviews and reporting; adapting processes to enable work to be done remotely – and only essential employees to be on site (remote data review, QC, QA, etc.); and managing quarantine and shutdowns in situations an employee tests positive for COVID-19. Simple tasks such as scanning of paper documents to enable remote access (for QC/QA) and the adoption of e-signatures for the approval of reports and regulatory documents became a necessity.

### Laboratory operations & safety

Work arrangements were changed to ensure the safety of the workers/employees including the use of different/multiple shifts to ensure social distancing and control the number of employees on site at a given time. This also necessitated the need for better communication and effective handoffs between the different shifts to avoid errors, missteps and avoid redundancy.

At the onset of the pandemic, there were questions around the safety of the workers and potential exposure resulting from the blood-based samples being analyzed. This was subsequently addressed by following existing guidelines on handling infectious blood samples (biosafety level 2, [BSL2]). In cases where nonclinical studies were performed using COVID infected animals, specific validated procedures for sterilization of processed samples became necessary to not only ensure that the samples could be safely shipped, but also safely received and appropriately handled at the bioanalytical lab upon arrival. Although, the time required for such a validated sterilization procedure was not trivial and did add to the overall timeline for bioanalysis, samples requiring BSL2 handling were at risk for not being able to be received into certain bioanalytical facilities.

Some bioanalytical facilities were never equipped to store or analyze larger numbers of BSL2-level samples prepandemic. COVID related sample analysis required BSL2 approved equipment for initial processing which imposed limitations on sample throughput. To mitigate such limitations, the sample processing steps of bioanalytical methods were validated to ensure specific stopping points could be incorporated to allow samples to be batched at a noninfectious stopping point to improve overall throughput and reduce worker stress. Safety teams at the analyzing labs shared concerns about the need for specific instrumentation and equipment to be ‘quarantined’ within their own sites, only for COVID use and only by certain personnel. These instruments, equipment and personnel would not be available for other work in the ‘nonquarantined’ lab space, which ultimately would be untenable. Consequently, it was imperative to remove this barrier to sample receipt and analysis from active COVID studies. Samples which were deemed noninfectious through a documented sterilization method could be more readily accepted and analyzed through the receiving lab’s standard processes.

### Additional stability experiments

Additional effort and experiments were needed to accommodate samples arriving at bioanalytical labs after long delays in transit or in customs, without dry-ice and partially/completely thawed. Such incidences required the conduct of additional stability experiments to establish stability and/or sample integrity.

### On site audits

From a sponsor perspective the ability to perform on-site visits or audits of the bioanalytical facilities and CROs was significantly impacted leading to canceling all formal visits. Alternatively, many of the CROs have implemented and welcomed virtual visits/audits. This has also impacted formal facility audits by the regulatory agencies, who have resorted to conducting virtual audits where most, but not all the documentation may be provided in a secure document sharing repository, supplemented with live/virtual participation including live video ‘tours/feeds’ when live viewing is needed.

## Impact on nonclinical trials

The supply of lab animals has been disrupted. US pharmaceutical companies have been scrambling to find cynomolgus monkeys. The shortage, caused by increases in demand for vaccine testing and a ban on shipments of wildlife from China, has caused some research projects to grind to a halt and inspired calls to maintain a reserve supply of animals [10]. As a separate option, movement to beagle dogs as the large animal species has been on the rise during late 2020 and continuing into 2021. While this has proved to be a viable alternative, the availability of beagles is now also under strain due to a lapse of breeding late in 2020, resulting in a delay of appropriately sized and aged dogs during 2021. Specific aspects of some therapeutics’ efficacy surrounding their evaluation in nonhuman primates cannot be substituted, so the issue of sourcing of cynomolgus monkeys or other related nonhuman primate species remains to be resolved even as the COVID study load decreases.

## Conclusion

The COVID-19 pandemic has provided both stressors and opportunities to the pharmaceutical and bioanalytical communities at large, many of which continue even now. For both, the existing processes were required to flex with the conflicting burdens of loss of staffing at facilities with added workloads for COVID related study support, drug development, bioanalysis and regulatory reviews in ultra rapid timeframes. Nonetheless, lessons can be taken from the overall outcomes, in that the innovative and highly collaborative efforts across teams within and outside of both pharma, bioanalytical labs and regulatory agencies worked together remarkably well. This was, of course, not without considerable rethinking of what was possible and removing barriers that hitherto had been considered immovable.

This was especially true when considering the interactions with regulatory agencies. Adapting to the situation at hand, what had been considered impossible became reality. It cannot be overstated how much the willingness to consider new options, adapt to new data and adjust planning to fit the needs of the population served was critical to the success of the various therapeutic and vaccine entities now available.

Even as the world continues to move toward a day in which the pandemic will reside in our past, we must consider the lessons learned from this period in time and hopefully utilize the best advances moving forward. Such adaptiveness can only provide better means and opportunities to provide the highly innovative and successful therapies sought by all within the pharmaceutical community.

## Future perspective

As the entire world grapples to step out from the COVID pandemic era, thanks to the development of vaccines and therapeutics and welcomes the ‘new normal’, the lessons learnt should be directed to mold our future – let’s continue to rethink processes and adopt innovative solutions and not revert back to the ‘same-old’ pre-COVID practices. From a drug (and vaccine) development perspective, the learnings are already dictating changes in not only how we work (i.e., telecommuting, hybrid work arrangements) but also how we have successfully adopted patient centric clinical practices (i.e., virtual doctor visits, mobile nursing, as well as self-sampling, patient centric blood sampling). Let’s not ignore or squander these valuable lessons and insights from this challenging period in history.

**Executive summary**

- Even as the world continues to move toward a day in which the pandemic will reside in our past, we must consider the lessons learned from this period in time and hopefully utilize the best advances moving forward.
- Creative and innovative adaptiveness can only provide better means and opportunities to provide the highly innovative and successful therapies sought by all within the pharmaceutical community.
